# Integrating sexual and reproductive health education with sports for young people: a global scoping review

**DOI:** 10.1186/s12889-026-26373-w

**Published:** 2026-02-10

**Authors:** Melissa N. Saphir, Marie K. Salem, Peggy Tahir, Vennela L. Devanaboyina, Martha J. Decker

**Affiliations:** 1 Philip R. Lee Institute for Health Policy Studies, University of California, San Francisco, 490 Illinois St, San Francisco, CA 94158 USA; 2 UCSF Library, University of California, San Francisco, 530 Parnassus Ave, San Francisco, CA 94143-0849 USA; 3 Department of Epidemiology and Biostatistics, University of California, San Francisco, 550 16th St, San Francisco, CA 94143 USA; 4 Bixby Center for Global Reproductive Health, University of California, San Francisco, San Francisco, CA USA

**Keywords:** Youth sports, Reproductive health, Health education, Scoping review, Global

## Abstract

**Background:**

Providing sexual and reproductive (SRH) health education to youth can improve outcomes including knowledge of sexually transmitted infections and use of condoms. Programs integrating soccer and sexual health education have shown promise in improving HIV-related outcomes in African countries; however, little is known about programs in other regions of the world, using other sports, or focusing on other SRH outcomes. Therefore, the purpose of this scoping review is to identify and compare a broader spectrum of integrated programs and their outcomes.

**Methods:**

Four databases were searched for articles mentioning SRH education, sports, adolescents, and synonyms of these concepts. References from articles selected for data extraction were also hand-searched. Articles were included in the review if they (1) reported on an intervention in which youth both play a sport and are explicitly taught a curriculum to improve any SRH outcome; (2) were published between 2000 and 2022; (3) reported quantitative outcome data; and (4) included participants between 10 and 24 years old. Two co-authors extracted data from the selected studies. Narrative synthesis and descriptive tables were used to summarize extracted data.

**Results:**

Of 4,161 records identified by the search, 21 met the inclusion criteria. The majority of the programs were implemented in Africa (*n* = 13). Sports included football/soccer (*n* = 15), netball/basketball (*n* = 2), cricket (*n* = 2), and unspecified sports (*n* = 4). Most (*n* = 12) interventions were multi-session programs lasting up to 12 weeks. HIV/AIDS prevention (*n* = 16) and prevention of gender-based violence (*n* = 13) were the most common SRH topics. Seven studies included random assignment to treatment and control. Significant improvements were reported by most of the studies measuring HIV-related outcomes as well as all three studies reporting outcomes related to contraception or pregnancy. Less than half of the studies measuring gender roles, gender norms, or gender-based violence reported positive outcomes.

**Conclusions:**

Integrated sport and SRH interventions show promise as a way to attract and engage youth in SRH programming. Additional research is needed to better understand the specific content, context, and implementation strategies that are associated with positive outcomes.

**Supplementary Information:**

The online version contains supplementary material available at 10.1186/s12889-026-26373-w.

## Background

Increasing young people’s access to quality sexual health education is critical to promote healthy sexual development, reduce negative health outcomes, and create a foundation for healthy adulthood. Globally, adolescents face pressing sexual and reproductive health (SRH) issues including dating violence, family planning, HIV and other sexually transmitted infections, and reproductive rights. However, youth in many countries lack access to clinic- or school-based SRH programming. Integrating sports and sexual health education is an innovative approach that has shown promise in African countries in improving outcomes related to HIV/AIDS [1]. Little is known about these types of programs more broadly, including programs conducted in other regions of the world and focusing on other SRH outcomes besides HIV/AIDS.

Programs promoting healthy development are increasingly using sports to facilitate health education for several reasons. Sports have historically been seen as inherently beneficial for physical and moral development [2, 3], and in 2003 the United Nations adopted a resolution calling on governments and agencies to promote sports “as a tool for health, education, and social and cultural development” [4]. Furthermore, because sports are engaging and fun for many youth, this can assist in program recruitment and participant retention [5].

Prior research differentiates between traditional and more integrated sport-based development programs [5, 6]. In traditional sports, healthy development, such as improved socioemotional outcomes, is assumed to follow as a benefit of playing sports. In the other approach, a health topic is an explicit part of the programming in addition to the sports component. Critical reviews assert that sport-based development programs are most effective when sport activities are combined with an explicit curriculum [2, 3, 5]. While an increasing number of programs have begun to integrate health promotion with a sports component, research on the topic remains limited, with poorly defined definitions of participation and outcomes [6].

SRH is a specific sub-field of this type of integrated programming that has emerged over the past few decades. While school-based SRH education has been shown to increase contraceptive use, decrease STI rates, and reduce teen pregnancy, many teens never receive this information [7]. In some settings, sports-based SRH education programs for young people may face fewer barriers to entry or may reach a different audience than school-based programs. For example, regular classroom teachers may be reluctant to teach about sex and HIV and may fear community disapproval, while coaches and out-of-school settings may have more flexibility [8]. Brady argues that integrating sports with other health and developmental content may be particularly important as a means of providing a safe space and empowering girls [9]. Given the significant need for new strategies to reach and engage youth to improve SRH outcomes [10], the feasibility and effectiveness of using sports warrants further investigation.

To date, most SRH-related research has focused on HIV/AIDS prevention, typically with soccer (also known as football) as the sport. SRH topics beyond HIV such as reproductive rights and contraception may have more relevance to youth needs, particularly in countries with low rates of HIV. In Hansell et al.’s 2021 review of 28 sport-based health promotion interventions in Africa, the top two most common categories of outcomes were physical fitness (measured by 9 programs) and HIV (8 programs) [11]. Of the programs identified, 14 used soccer, with other programs using ropes courses, playground games, netball (basketball), dance, and stretching. Kaufman et al.’s 2013 meta-analysis of 21 sport-based HIV prevention programs worldwide found that these programs improved HIV-related outcomes including knowledge, attitudes, self-efficacy, reported recent condom use, and communication about HIV but did not significantly increase uptake of HIV-related counseling or testing [1]. In this review, more than two thirds of the reviewed interventions consisted of curricula incorporating sports themes, metaphors, or in-class activities or consisted of a health curriculum delivered by an athlete. In other words, most of the reviewed HIV interventions involved little or no sports participation by the youth receiving the curricula.

Neither Hansell et al. nor Kaufman et al. specifically focused on programs in which SRH programming is integrated with youth playing sports. The purpose of this scoping review is to fill this gap and expand the breadth of programming reviewed by (1) identifying programs that engage youth in playing sports and also provide educational content to improve SRH outcomes and (2) summarizing the results for the full variety of programs that have been assessed globally. This information can inform future program design and implementation in other settings and with additional populations.

## Methods

### Study design

This review followed recommended methods for scoping reviews [12] and adhered to the Preferred Reporting Items for Systematic Reviews and Meta-Analysis Extension for Scoping Reviews (PRISMA-ScR) [13]. A date-stamped version of the registered protocol is available via the Open Science Foundation (https://osf.io/d2fht).

The review was designed to answer the question: “What are the SRH outcomes of youth programs that integrate sports and SRH interventions?” A research librarian was consulted to construct searches and advise on database selection. Four databases were searched on October 28, 2022: PubMed, Embase, Web of Science, and Sociological Abstracts. Searches were developed to be broad and inclusive, and included both keywords and index terms (i.e., medical subject headings (MeSH) or Emtree vocabulary), as appropriate for each database. Abstracts and articles in English, Spanish, and Portuguese were reviewed. The main concepts considered were sexual health education, sports, and adolescents. Multiple synonyms were developed for each concept. Full search strategies for each database are included in Supplemental Table 1. We also reviewed the gray literature by hand-searching references from articles selected for data extraction. Articles were included in the review if they met the following criteria:

1. Published between January 1, 2000, and October 26, 2022.
2. Reported quantitative outcome data.
3. Reported on an integrated sport and SRH intervention program.
4. Included participants between the ages of 10 and 24 years old (i.e., the World Health Organization’s definition of young people [14]).

Studies that met al.l these criteria were excluded if they reported on the same data that had previously been reported in another publication. For ease of interpretation and to facilitate comparison of outcomes across studies, qualitative studies, review articles, theoretical articles, pilot studies, posters, abstracts, and protocols were also excluded.

### Study selection and coding

EndNote software version 20.4 [15] was used to remove duplicates from the lists of articles returned by the database searches. Of the resulting preliminary list of articles, two research assistants independently reviewed the titles and abstracts to determine whether each report warranted further review. They then reviewed the full text of the articles passing the title/abstract review to determine which articles met the inclusion criteria. The research assistants consulted with the principal investigator to resolve any disagreements and clarify inclusion criteria.

The full text of the final set of included articles was read in full by two authors. One author extracted data from the articles, and a second author verified the accuracy of the extracted information. Extracted data were recorded on a form developed by two of the authors and included the country (or countries) of program implementation, description of participants (age, gender, race/ethnicity, and other relevant demographic characteristics), the study’s design (e.g., randomized clinical trial, quasi-experiment) and funding source (e.g., national government, private foundation, sport organization, international organization, or university), description of intervention program (type of sport, educational component, program length/dosage, setting), the outcomes that were measured, and the study findings.

### Analyses

The goal of any scoping review analysis is primarily descriptive, in this case, to summarize the range and types of integrated sports and SRH intervention programs and the SRH outcomes that have been studied, synthesize the results, and ascertain gaps in the existing research. This review therefore used narrative synthesis supported by descriptive tables that summarize data extracted from the included studies, as recommended by Arksey and O’Malley [12] and Popay et al. [16].

## Results

**Fig. 1:**
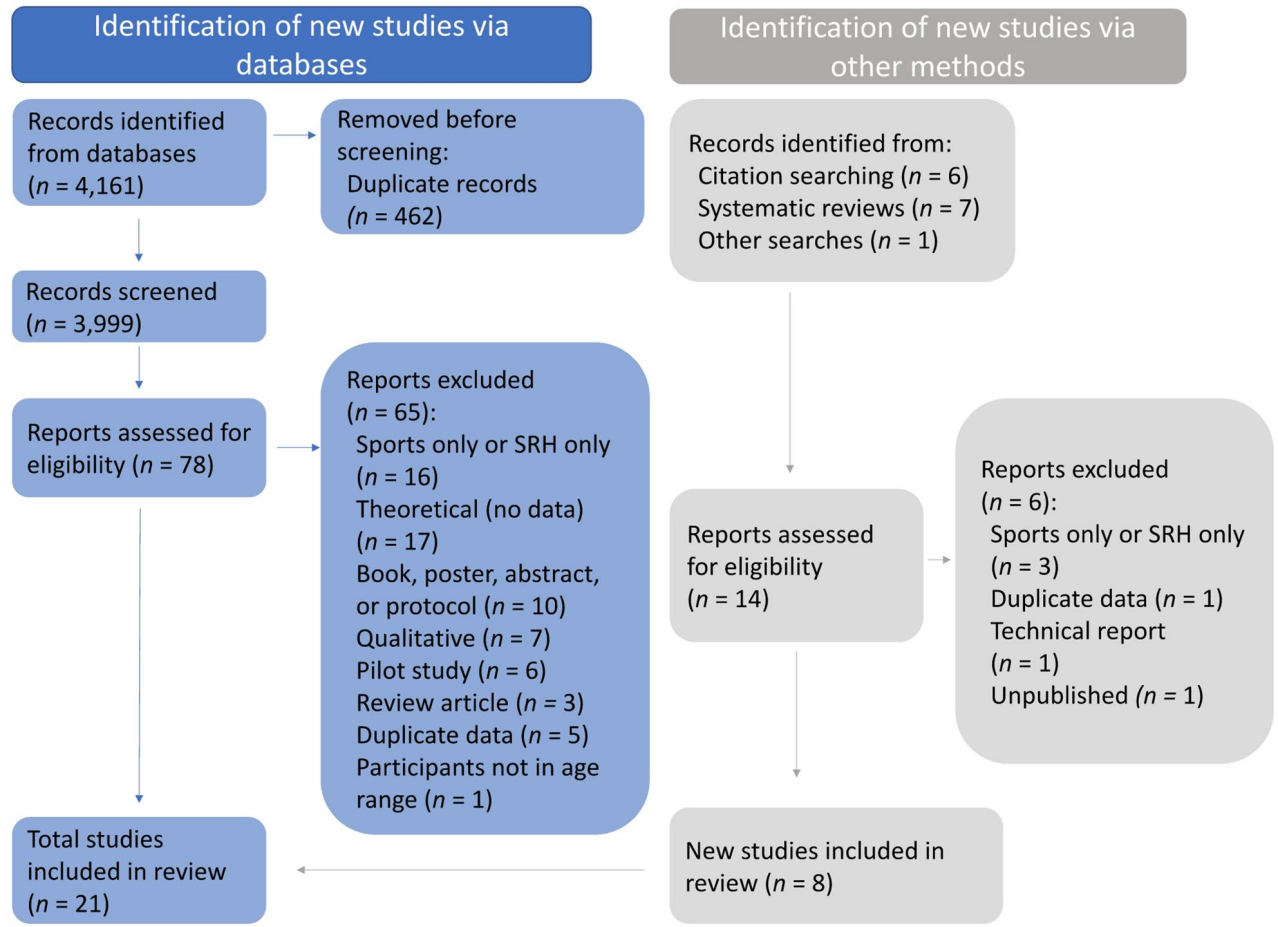
PRISMA article identification and selection flow diagram

The database searches identified 4,161 records, from which 462 duplicates were removed. Title and abstract screening was conducted on the remaining 3,999 records (see Fig. 1). Of these, 78 studies and an additional 14 studies identified by citation searching and review articles were read in full to assess their eligibility. A total of 21 studies met the inclusion criteria. Table 1 provides details about each included study’s SRH educational component, sport(s), participants, the country (or countries) in which the programs were administered, and the study’s design and funding source(s). Table 2 summarizes several of these characteristics.

**Table 1:** Characteristics of programs incorporating sports and SRH education (*N* of reviewed studies = 21)

First author (year)	Educational component	Who taught lessons?	Sport	Duration of program	Partici-pants	Country	Study design	Funding source
Awotidebe et al. (2014) [25]	HIV prevention activities to promote knowledge of risks and self-efficacy to abstain from sex and resist peer pressure	Peer educators	Soccer	11 sessions over 12 weeks	340 students in grades 8–10	South Africa	Quasi-experi-ment	University
Balfour et al. (2013) [26]	HIV prevention activities to change attitudes toward HIV and promote self-efficacy to make healthy choices	Counselors	Soccer	Eight 90-min sessions over 12 weeks	629 students in grades 5–8	South Africa	Quasi-experi-ment	National government
de Carcer (2020) [23]	Lessons on HIV/AIDS, malaria, hygiene, and leadership combined with soccer-based games	Coaches and program leaders	Soccer	Single 90-minute session	120 youth ages 9–16 years	Cameroon	RCT	Sport organization
Delva et al. (2010) [27]	HIV/AIDS prevention education given to existing teams; education plus movement games at schools; prevention information provided at tournaments.	Peer educators	Unknown	Varied from 10 min to 3 h. Youth chose how much to participate.	772 youth ages 12–24 years	Kenya	Quasi-experi-ment	Not reported
Duffey et al. (2019) [32]	Lessons on sexuality, SRH, sexual and reproductive rights, sexual violence, pregnancy, and menstruation provided to girl players and spectators.	Coaches	Soccer	Varied from none to 2 years.	120 girls ages 12–24 years	Zambia	Correla-tional (cross-section-al survey)	Sport organization
Fuller et al. (2010) [24]	Coaching in soccer skills followed by prevention information about disease and health conditions.	Coaches	Soccer	90 min (probably 11 sessions)	370 students in grades 6–7	South Africa	Quasi-experi-ment with random assign-ment of schools	Sport organization
Fuller et al. (2011) [28]	Coaching in soccer skills followed by prevention information about disease and health conditions.	School teachers in Mauritius; coaches in Zimbabwe	Soccer	90 min (probably 11 sessions)	389 youth ages 12–15 in Mauritius; 395 youth ages 10–14 in Zimbabwe	Mauritius, Zimbabwe	Pre-post, no control	Sport organization
Fuller et al. (2015) [29]	Coaching in soccer skills followed by prevention information about disease and health conditions.	School teachers	Soccer	Eleven 90-min sessions	1,555 youth ages 9–12 years	Brazil	Pre-post, no control	Sport organization & foundation
Hershow et al. (2015) [33]	Lessons after school integrating soccer, life skills activities, and counseling and testing to prevent HIV/AIDS.	Female community leaders	Soccer	Ten 2-hr sessions	514 girls ages 11–14 years	South Africa	Pre-post, no control	National government & foundation
Jejeebhoy et al. (2017) [18]	Lessons on gender roles, violence against women, respectful behavior towards women, and positive masculinity. Periodic community events promoted gender egalitarian attitudes.	Peer mentors overseen by trainers & coaches	Cricket	Forty-two 2-hr sessions over 18 months	1,149 boys ages 13–21 years	India	Cluster RCT	National government
Kaplan et al. (2015) [34]	Program to support SRH education and change gender norms included: 1) SRH courses, 2) Girls’ Health Days, 3) all-female summer soccer league, and 4) World AIDS Day community event	Community health workers	Soccer	Varied: yearlong program of five 2-hr SRH sessions in 1 week; daily summer soccer practice	4,251 girls ages 15–19 years	Haiti	Quasi-experi-ment	Foundation
Kaufman et al. (2012) [19]	Interactive sport activity combined with discussion of health HIV-related risks.	Community soccer players	Soccer	10 h over 5 days	140 youth, median age 14 years	Dominican Republic	Cluster RCT	Foundation
Kaufman et al. (2016) [30]	Interactive game, personal story shared by coach, and group discussion to increase voluntary male medical circumcision.	Circumcised adult male facilitators	Soccer	Single 60 min session	1,226 boys ages 14–20 years	Zimbabwe	Quasi-experi-ment	International organization
Marcus & Stavropoulou (2020) [17]	Lessons about health (including SRH), communication, rights, and managing personal finances.	Varied: facilitators from implementing organizations and peer leaders	Varied by country, including netball (basketball), soccer, cricket	Weekly sessions for 10 months	18,698 girls ages 12–18 years	India, Kenya, Myanmar, Nigeria, Pakistan, South Africa, Uganda Zambia	Pre-post, no control	International organization
Maro et al. (2009) [35]	AIDS education alongside soccer games.	Peer coaches	Soccer	8 weeks (frequency and length of sessions unknown)	764 youth, average age 13.6 years	Tanzania	Quasi-experi-ment	Not reported
Merrill et al. (2018) [31]	Lessons on body image, SRH and HIV knowledge, and decision-making in relationships. Texting service reinforced curriculum.	Female community leaders	Soccer	Ten 2-hr sessions over 5 weeks	213 girls ages 11–16 years	South Africa	Pre-post, no control	Foundation
Miller et al. (2012) [20]	Brief discussions about respect and dating violence prevention.	School coaches	Unknown, multiple sports	Eleven 10- to 15-min discussions	1,798 boys in grades 9–12	United States	Cluster RCT	National government
Miller et al. (2020) [21]	Brief discussions about (1) respectful relationship behaviors, (2) gender-equitable attitudes, and (3) positive bystander intervention.	School coaches	Unknown, multiple sports	Twelve 15-min discussions over 12 weeks	973 boys in grades 6–8	United States	Cluster RCT	National government
Sieverding & Elbadawy (2016) [37]	Lessons in literacy and life skills, reproductive health, gender roles, and sport were taught in safe spaces. Family members also received some education.	Women with secondary education	Unknown	12 h per week for 20 months	2,248 girls ages 10–16 years	Egypt	Quasi-experi-ment	International organization & national government
Tingey et al. (2015) [22]	Training in condom use and lessons on SRH behaviors, knowledge, attitudes, perceptions, beliefs, practical skills, and intentions to prevent HIV/AIDS, culturally customized for American Indian youth.	American Indian para-professionals ages 25–50	Basketball	4-hr sessions over 8 consecutive days	267 youth ages 13–19 years	United States	Cluster RCT	National government
Woodcock et al. (2012) [36]	Lessons on SRH, HIV prevention, and economic and individual empowerment.	Peer educators	Soccer	Years-long program, dose varied	333 girls, ages 10–19 years	Kenya	Cross-section-al	Foundation

### Research designs

Over a third of the reviewed studies (*n* = 8) reported data on fewer than 500 youth participants (Table 2). Six studies reported data on 500 to 1,000 participants, another six reported on 1,000 to 5,000 participants. The largest study, with over 18,000 participants, was an evaluation of a program implemented in nine countries [17].

Many different research designs were used by the reviewed studies, but the majority had either no control group (*n* = 7) or no random assignment to treatment and control groups (*n* = 7). Of the seven studies with a control group and random assignment, five were cluster randomized controlled trials (RCTs) [18–22], and one was an RCT with randomization at the individual level [23]. One study initially randomized two schools to the intervention or control group and then subsequently added another intervention group within the same school [24].

The most common funding sources for the studies of these programs were grants from national governments (*n* = 7), foundations (*n* = 6), and sports organizations (*n* = 5). All five studies funded by a sports organization were evaluations of soccer programs. Two studies did not report a source of funding.

### Characteristics of integrated sports and SRH programs

The majority of the programs were conducted in Africa, with multiple programs in South Africa (*n* = 6), Kenya (*n* = 3), Zambia (*n* = 2), and Zimbabwe (*n* = 2). Three programs were conducted in the United States, and three were conducted in the Latin American and Caribbean countries of Brazil, the Dominican Republic, and Haiti. Two programs were conducted in Asia, including one in India and one in multiple countries (India, Myanmar, and Pakistan).

Almost three quarters of the integrated sport and SRH intervention programs (*n* = 15) employed soccer (Table 2). The only other sports mentioned were cricket (*n* = 2), netball (*n* =1), and basketball (*n* = 1). Three studies were based on multiple sports, and four studies did not state the sport. The studied interventions were slightly more likely to have youth engage in sport-themed activities (such as training in sports skills or unspecified physical games) (*n* = 12) [19, 22–31] than in full matches (*n* = 9) [17, 18, 20, 21, 32–36]. Interventions in which youth played full matches were connected with a soccer league or with a school’s competitive athletics program.

**Table 2:** Characteristics of included programs (*N* of reviewed studies = 21)

Characteristic	*N* (%)
Sport^a^	
Soccer (football)	15 (71%)
Basketball or netball	2 (10%)
Cricket	2 (10%)
Multiple sports	3 (14%)
Unknown	4 (19%)
Duration of program^b^	
1 day	3 (14%)
More than 1 day, up to 12 weeks	12 (57%)
More than 12 weeks, up to 1 year^a^	2 (10%)
More than 1 year^a^	4 (19%)
Extent of youth participant in sport	
Sport-themed activities	11 (52%)
Played full matches	9 (43%)
Unknown	1 (5%)
Family or community members received educational programming	4 (19%)
Sex of participants	
All included	10 (48%)
Girls only	7 (33%)
Boys only	4 (19%)

^a^Percentages do not sum to 100% because some studies appear in multiple categories

^b^In longer programs, duration varied depending on participants’ desire to continue

The shortest interventions consisted of a single 60- or 90-minute session (*n* = 3), but the majority (*n* = 12) were multi-session programs lasting as long as 12 weeks. Six interventions continued for a year or more. While most of the programs provided a specific “dose” of sport and SRH instruction to all participants, the four longest interventions [27, 32, 34, 36] gave participants latitude to choose how much time to spend in the program. For example, the SRH program evaluated by Duffey et al. [32] provided SRH educational sessions for girl soccer players for up to two years, with the players choosing how long to continue playing with the league and receiving the lessons.

A third of the reviewed studies (*n* = 7) included only girls as participants, four studies included only boys, and the remainder (*n* = 10) included girls and boys. Single-sex interventions tended to have a sex- or gender-specific goal. For example, three of the four programs for boys focused in part on cultivating respect for women and girls and preventing violence toward women [18, 20, 21]. The fourth program that included only boys focused on increasing voluntary male medical circumcision [30]. All seven of the programs for girls included lessons on women’s rights or empowerment.

In addition to educating youth participants, four interventions included components in which educational programming on SRH topics was provided to community members or family members. Several programs disseminated their educational content to the broader community, by conducting community events [18, 34], providing information to the audience during the soccer matches of the participating youth [27], by sending mentors to participants’ homes to educate family members [37].

Most of the education components of the integrated programs included more than one SRH topic. The most common topic was HIV/AIDS prevention (*n* = 16). The second most common topic was prevention of gender-based violence (*n* = 13), followed by gender roles and norms and/or girl empowerment (*n* = 11). Note that all but one [33] of the programs focused on gender norms or empowerment also included gender-based violence as a topic. General SRH was a focus of eight studies, including non-specific references to sexual health, reproductive health, SRH, or sexual anatomy. Relationships, communication, or sexual negotiation were a topic of eight studies. Four studies focused on contraception and/or pregnancy, and three studies focused on encouraging youth to get SRH health care. The single most common topic not directly related to SRH was substance use (*n* = 5). Six studies included components related to other non-sexual health topics such as malaria prevention, diet, exercise, and hygiene.

### Outcomes

Table 3 summarizes the SRH outcomes reported by the reviewed studies. (Note that studies did not always report or measure outcomes for every topic their educational programs discussed.) Similar to the intervention topics, the most commonly reported outcomes were HIV-related. Five studies reported on HIV-related sexual behavior, including condom use, having an exclusive partner, risky sexual behavior, and male circumcision. Of these, significant main effects were reported for condom use [22, 32, 35], having an exclusive sexual partner [35], and male circumcision [30]. One reported no improvement in risky sexual behavior [25], and one reported mixed results for condom use, number of sexual partners, and having concurrent relationships [27]. Fourteen studies reported other HIV-related outcomes, including knowledge (e.g., about how to prevent HIV transmission), attitudes, beliefs, self-efficacy, norms, or communication (e.g., negotiating with partners about condoms). Of these studies, eight reported significant improvement in the other HIV outcomes, two reported no significant improvement, and two reported mixed results. Two of these studies reported improvements in HIV-related outcomes but did not conduct significance testing [29, 31].

**Table 3:** Selected SRH outcomes, by study

Author (year)	HIV (sexual behavior)	HIV (other outcomes)	Gender roles/norms	Gender violence	Contraception/ pregnancy
Awotidebe et al. (2014) [25]	N	Y			
Balfour et al. (2013) [26]		Y			
de Carcer (2020) [23]		M			
Delva et al. (2010) [27]	M	N			
Duffey et al. (2019) [32]	Y	Y			
Fuller et al. (2010) [24]		N		N	
Fuller et al. (2011) [28]		Y	Y		
Fuller et al. (2015) [29]		Y^1^	Y^1^		
Hershow et al. (2015) [33]		Y	N		
Jejeebhoy et al. (2017) [18]			Y	N	
Kaplan et al. (2015) [34]					Y
Kaufman et al. (2012) [19]		Y			
Kaufman et al. (2016) [30]	Y				
Marcus & Stavropoulou (2020) [17]				Y	Y
Maro et al. (2009) [35]	Y	M			
Merrill et al. (2018) [31]		Y^1^	Y^1^	Y^1^	
Miller et al. (2012) [20]			N	N	
Miller et al. (2020) [21]			N	N	
Sieverding & Elbadawy (2016) [37]			Y	Y	Y
Tingey et al. (2015) [22]		Y			
Woodcock et al. (2012) [36]		Y			

Notes: A blank cell indicates the outcome was not measured. *Y* indicates the main effect for the *only* measure of concept was significant at *p* < 0.05, or main effects for *all* measures of the concept were significant. *N* indicates the main effect for the *only* measure of concept was NOT significant, or main effects for *all* measures of the concept were not significant. *M* indicates mixed results (i.e., the main effect for one measure of concept was significant, but main effects for other measures were not significant; *or* the only significant effect was in a subpopulation

^1^There was no significance testing

Of the eight studies that reported outcomes related to gender roles or norms, three reported significant main effects. An 11-week soccer and health education program resulted in improvements in gender-related attitudes among students in grades 6 and 7 in South Africa [28]. A 20-month program called Ishraq that included SRH education and unspecified sports for Egyptian girls increased participant agreement that girls should be 18 years old or older before getting married (a norm intended to reduce child marriage) [37]. An 18-month gender skills education program integrated with cricket for male youth in India significantly improved gender norms and attitudes toward masculinity among participants [18]. Two additional studies reported improvements in attitudes toward gender equality but did not conduct statistical testing [29, 31]. Three studies [20, 21, 24, 33] found no significant changes in gender-related outcomes.

Of the seven studies that reported outcomes related to gender violence, two reported significant main effects. A program called Goal that taught SRH information and girl empowerment alongside sports was evaluated in eight countries and was found to increase resistance to gender-based violence, knowledge of how to get help for such violence, and rejection of the attitude that such violence is acceptable [17]. The Ishraq program in Egypt also significantly reduced girls’ intention to perform female genital cutting on their future daughters [37]. One additional study reported improvement in knowledge of where to get services after rape but did not conduct statistical testing [29, 31]. No significant changes in gender-based violence outcomes were found by four studies [20, 21, 24, 33], including the Indian cricket program described above that improved gender equality attitudes [18].

Only three studies reported on outcomes related to contraception or pregnancy, and all three reported significant improvements. Haitian teens who participated in an SRH education program that was integrated with a girls-only summer soccer league had a lower birthrate than non-participants 10 years after the program [34]. Notably, girls who participated in both the education and the sport components of the Haitian program had significantly lower birth rates than those who only received the education component, who in turn had significantly lower birth rates than non-participants. The Ishraq program also increased knowledge about contraceptives and reduced the number of children desired by the female participants [37]. The eight-country evaluation of the Goal program also found significant increases in girls’ knowledge on a health measure that included knowledge of how to prevent unwanted pregnancy [17].

The five studies funded by sports organizations reported positive outcomes in a total of six categories out of a total of nine categories of outcomes reported (67%). The 16 studies funded by other types of organizations had a similar likelihood of reporting positive outcomes (63%).

Comparison of Tables 1 and 3 reveals that positive outcomes were not limited to a particular sport. That is, 13 of the 15 studies employing soccer reported positive and/or mixed outcomes, as did all three of the studies employing other sports (cricket and/or netball). Similarly, positive outcomes were found from interventions with varying levels of sports engagement by youth — that is, programs in which participants played full matches and in which participants engaged in sport-themed activities but did not compete.

## Discussion

This scoping review identified 21 evaluations of programs around the world that integrated youth sports and SRH education. As found in prior reviews of youth sport and health interventions [1, 11], most were based in Africa and used soccer as the sport. However, we did identify several programs implemented in other parts of the world and programs that used other sports such as cricket, netball, and basketball. Unlike prior reviews, we also identified a substantial number of programs that focused on content beyond HIV, such as healthy relationships and access to SRH services. All three of the programs focused on contraception showed positive outcomes. Programs focused on HIV-related outcomes, whether they were changes in sexual behaviors or related knowledge and attitudes, were the next most likely to report positive outcomes. Over half of the interventions included topics related to gender norms, with many all-girl programs emphasizing girls’ empowerment. However, these programs showed mixed results in outcomes related to gender norms and gender-based violence. Further research is needed to explore the interplay between different topics, types of sport, and youth populations to determine whether some are a better fit with this type of integrated programming. In addition, the complexity of the content covered may affect the reported outcomes.

Most of the programs identified by this review prioritized youth who face greater barriers than other youth to participation in sports as well as to receipt of SRH information and services and were implemented in low-resource settings. For example, many of the programs prioritized youth from underserved groups including youth from rural areas [22, 25, 34, 37] and disadvantaged communities [17, 27]. In many instances, youth from low-income communities and other vulnerable groups face greater barriers to participation in sports as well as barriers to SRH information and services [38–40]. Similarly, several of the programs prioritized girls [32–34], who are less likely to engage in sports or physical activity compared to boys worldwide [41, 42]. This type of integrated programming is a promising means of engaging these populations in both needed SRH content and sports that they may not receive elsewhere. In addition to improving SRH outcomes, prior research has found that sports can also improve developmental outcomes such as self-regulation among socially vulnerable youth [43].

The programs identified by this review incorporated a range of approaches, topics, and study designs, thus making comparisons across studies challenging. In addition, the programs employed a variety of individuals to teach the SRH components, ranging from peer coaches to schoolteachers. Previous research regarding sports and positive youth development programming stressed the importance of program context such as the relationships between the youth participants and other peers and adults on outcomes [44, 45]. Further research is needed to determine what type of training can support facilitators to be comfortable in discussing sensitive topics and creating a supportive environment as well as in effective coaching techniques. Similarly, few of the articles provided detail about fidelity or other related implementation factors that may affect program outcomes. A future scoping review that includes qualitative studies may provide additional detail into the context of the programs as well as participant and staff perspectives. Additionally, future research should explore the effectiveness of other approaches that use sports as an entry to provide SRH education and information, such as information sessions for spectators during matches.

We did not find evidence that a specific sport or extent of sports participation was necessary for positive outcomes. This suggests that other sports and a range of participatory approaches may be appropriate. Since youth vary in their interest in soccer, expanding the range of sports employed by SRH interventions may increase the type and number of youth who receive these promising interventions. In addition to the programs included in this review, we identified several interventions that used other sports but did not meet the required inclusion criteria. This included one program in the United States where youth designed and performed step-dancing routines that imparted SRH public service announcements [46] and a program integrating hatha yoga and adherence counseling for adolescents with HIV in India [47]. These innovative programs may be of interest in future research and program development.

The results of this scoping review had some limitations. Importantly, four did not specify the sport played, and several did not report other key program characteristics, making cross-program comparisons challenging. In addition, fewer than half of the studies identified were RCTs, limiting the causal inferences that can be drawn from the evaluations. Only one study was published in a language other than English (Spanish), despite searching for studies in Spanish and Portuguese, which may reflect a language bias in published research. Despite these limitations, this review successfully identified programs across world using different sports and measuring distinct SRH outcomes.

Designers and implementers of SRH interventions for youth should be encouraged by the results of this scoping review to employ sports as a way to attract youth to their programs and keep them engaged in SRH education. Future programs should consider additional types of sports to reach different youth populations and consider the implementation context, including the quality of the coaching and fidelity to the programming. Future evaluation research should clearly describe their curricula and sport components, so that programs can be compared and effective components replicated more easily.

## Conclusions

Integrated sports and SRH interventions show promise to improve SRH outcomes among youth in a variety of settings and with different sports programming. Programs focusing on contraception and HIV-related outcomes showed more consistent positive results than those measuring gender norms or gender-based violence. Further research is needed to better understand the specific content, context, and implementation strategies that are likely to account for improved outcomes.

## Supplementary Information

Supplementary Material 1

## Data Availability

No datasets were generated or analysed during the current study.

## Abbreviations

HIV: Human immunodeficiency virus
AIDS: Acquired immunodeficiency syndrome
hr: Hour
MeSH: Medical subject heading
min: Minute
PRISMA-ScR: Preferred Reporting Items for Systematic Reviews and Meta-Analysis Extension for Scoping Reviews
RCT: Randomized controlled trial
SRH: Sexual and reproductive health
