# Supplementary material for: Integrating sexual and reproductive health education with sports for young people: a global scoping review
Source: BMC Public Health. 2026 Feb 10;26:896. doi: 10.1186/s12889-026-26373-w (PMC12998158; doi:10.1186/s12889-026-26373-w)
Supplement: Supplementary file 1 — Supplementary Material 1 [file 12889_2026_26373_MOESM1_ESM.docx]

Supplemental Table 1. Search strategy for each database

| **Database** | **Search strategy** |
| --- | --- |
| PubMed | (“Sex Education”[MeSH] OR “sex education”[tiab] OR “Pregnancy in Adolescence”[MeSH] OR “adolescent pregnancy”[tiab] OR “teen pregnancy”[tiab] OR “Reproductive Health Services”[MeSH] OR “reproductive health”[tiab] OR “HIV Infections/prevention and control”[MeSH] OR HIV[tiab] OR “Acquired Immunodeficiency Syndrome/prevention and control”[MeSH] OR “acquired immunodeficiency syndrome”[tiab] OR “Sexual Behavior”[MeSH] OR “sexual behavior”[tiab] OR “School Health Services”[MeSH] OR “school health services” OR “sexual risk”[tiab] OR contraception[tiab] OR “Contraception”[MeSH]) AND ("Sports"[Mesh] OR sport[tiab] OR sports[tiab] OR “organized sports”[tiab] OR soccer[tiab] OR football[tiab]) AND (“Adolescent”[MeSH] OR adolescent[tiab] OR adolescence[tiab] OR “Young Adult”[MeSH] OR “young adult”[tiab] OR youth[tiab] OR teen[tiab] OR teens[tiab] OR teenagers[tiab] OR “Child”[MeSH] OR child[tiab] OR children[tiab]) |
| Web of Science | (“sex education” OR “adolescent pregnancy” OR “teen pregnancy” OR “reproductive health” OR HIV OR “acquired immunodeficiency syndrome” OR “sexual behavior” OR “school health services” OR “sexual risk” OR contraception) AND (sport OR sports OR “organized sports” OR soccer OR football) AND (adolescent OR adolescence OR “young adult” OR youth OR teen OR teens OR teenagers OR child OR children) |
| Embase | ('sexual education'/exp OR 'sexual education' OR 'adolescent pregnancy'/exp OR 'adolescent pregnancy' OR 'adolescent pregnancy prevention and control'/exp OR 'adolescent pregnancy prevention and control' OR 'reproductive health'/exp OR 'reproductive health' OR 'hiv infections prevention and control'/exp OR 'hiv infections prevention and control' OR 'human immunodeficiency virus infection'/exp OR 'human immunodeficiency virus infection' OR 'acquired immunodeficiency syndrome prevention and control'/exp OR 'acquired immunodeficiency syndrome prevention and control' OR 'acquired immune deficiency syndrome'/exp OR 'acquired immune deficiency syndrome' OR 'sexual behavior'/exp OR 'sexual behavior' OR 'school health service'/exp OR 'school health service' OR 'sexual risk'/exp OR 'sexual risk' OR 'sexual risk behavior'/exp OR 'sexual risk behavior' OR 'contraception'/exp OR contraception) AND ('sport'/exp OR sport OR 'organized sports' OR 'soccer'/exp OR soccer OR 'football'/exp OR football) AND ('adolescent'/exp OR adolescent OR 'adolescence'/exp OR adolescence OR 'young adult'/exp OR 'young adult' OR 'juvenile'/exp OR juvenile OR 'child'/exp OR child) AND [2000-2021]/py |
| Sociological Abstracts | (“sex education” OR “adolescent pregnancy” OR “teen pregnancy” OR “reproductive health” OR HIV OR “acquired immunodeficiency syndrome” OR “sexual behavior” OR “school health services” OR “sexual risk” OR contraception) AND (sport OR sports OR “organized sports” OR soccer OR football) AND (adolescent OR adolescence OR “young adult” OR youth OR teen OR teens OR teenagers OR child OR children) |

Notes. “[tiab]” indicates that the search term must appear in the title or abstract.
